# Epidemiology of colorectal cancer: A review with special emphasis on India

**DOI:** 10.1007/s12664-024-01726-8

**Published:** 2025-02-10

**Authors:** Samyukta Shivshankar, Prachi S. Patil, Kedar Deodhar, Atul M. Budukh

**Affiliations:** 1 Homi Bhabha National Institute, Training School Complex, Anushakti Nagar, Mumbai, 400 094 India; 2 Division of Medical Records and Cancer Registries, Centre for Cancer Epidemiology, Advanced Centre for Treatment, Research and Education on Cancer, Tata Memorial Centre, Kharghar, Navi Mumbai, 410 210 India; 3 Department of Digestive Diseases and Clinical Nutrition, Tata Memorial Hospital, Dr Ernest Borges Road, Mumbai, 400 012 India; 4 Department of Pathology, Tata Memorial Hospital, Dr Ernest Borges Road, Mumbai, 400 012 India

**Keywords:** Colorectal cancer, Epidemiology, Incidence, India, Risk factors

## Abstract

Colorectal cancer (CRC) is a common malignancy and cause for death around the world. In India, it ranks as the fourth most incident cancer in both sexes, with 64,863 cases and 38,367 deaths in 2022. With such high mortality, CRC survival in India is way lesser than that of developed countries. While western countries are facing an overall decline in CRC incidence, various regions in India are seeing an increasing trend. Within India, urban regions have markedly higher incidence than rural. Risk factors include consumption of red and processed meat, fried and sugary food, smoking and alcohol, comorbidities such as obesity, diabetes and inflammatory bowel disease (IBD), family history of CRC, adenomas and genetic syndromes, radiation exposure, pesticides and asbestos. Consumption of nutrient-rich well-balanced diets abundant in vegetables, dairy products, whole grains, nuts and legumes combined with physical activity are protective against CRC. Besides these, metformin, aspirin and micronutrient supplements were inversely associated with the development of CRC. Since a considerable proportion of CRC burden is attributed to modifiable risk factors, execution of population level preventive strategies is essential to limit the growing burden of CRC. Identifying the necessity, in this review, we explore opportunities for primary prevention and for identifying high-risk populations of CRC to control its burden in the near future.

Colorectal cancer (CRC) is the third most incident type of cancer, worldwide, following lung and prostate in males and breast and lung in females [1]. Over 1.9 million CRC cases (males:1,045,413; females:826,706) occurred globally in 2022, with an age adjusted rate per 100,000 (AAR) of 17.8 [1]. Additionally, 881,984 deaths occurred due to CRC (mortality AAR 7.8) [1]. CRC contributes to more than 9% of the world’s cancer incidence and mortality [1].

As per the international classification of diseases (ICD-10), this anatomical grouping includes colon (C18), rectosigmoid junction (C19) and rectum (C20) [1–3]. CRC is common among males and older age groups [4–6]. European, Oceanian and North American countries have the highest incidence of CRC in the world [4]. Within continents, the difference in CRC incidence between high income and low and middle-income countries (LMICs) is striking [1, 2], as shown in Table 1.

**Table 1:** Areas with highest and lowest incidence of colorectal cancer for 2013–17 from cancer incidence in five continents XII

Continent	Males				Females			
	Highest incidence		Lowest incidence		Highest incidence		Lowest incidence	
	Country, area	Number (AAR per 100,000)	Country, area	Number (AAR per 100,000)	Country, area	Number (AAR per 100,000)	Country, area	Number (AAR per 100,000)
Africa	La Réunion (French Territory)	659 (30.8)	Uganda, Gulu	10 (1.9)	La Réunion (French Territory)	604 (22.5)	Uganda, Gulu	6 (1.2)
Asia	Japan, Aomori	5885 (67.0)	India, Sangrur	63 (2.3)	Japan, Akita prefecture	2213 (36.5)	India, Sangrur	38 (1.5)
Europe	Spain, Navarra	1464 (56.9)	Switzerland, Lucerne	695 (27.1)	Norway	10,726 (37.6)	Austria, Carinthia	614 (15.9)
Central and South America and the Caribbean Islands	Uruguay	4704 (36.6)	Ecuador, Manabi	305 (8.6)	Uruguay	4480 (25.7)	Ecuador, Manabi	286 (7.7)
North America	Canada, Newfoundland	1678 (58.8)	USA, North Carolina, Asian and Pacific Islanders	113 (15.7)	USA, Alaska, Alaskan natives	219 (60.3)	USA, Ohio, Asian and Pacific Islanders	99 (11.6)
Oceania	Australia, Tasmania	1201 (44.6)	Australia, Northern territory (indigenous)	20 (14.9)	Australia, Northern territory (non-indigenous)	166 (34.5)	Australia, Northern territory (indigenous)	20 (12.5)

AAR is age adjusted rate per 100,000

India has traditionally been considered a low incidence country. However, there is a recent perception among clinicians of a rising trend especially of young onset CRC in India [7]. In this article, we present the epidemiology of CRC in India through latest reports and studies on incidence, trends, mortality, survival and risk factors.

## Colorectal cancer incidence in India

**Fig. 1:**
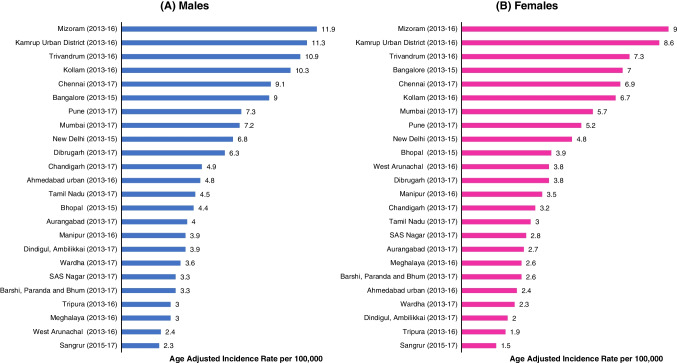
Comparison of colorectal cancer incidence in India in 2013–17 using data from Cancer Incidence in Five Continents XII;link: https://ci5.iarc.fr/ci5-xii/tables/by-site-3-digits

Colorectum ranks among the most common sites of cancer incidence in India, with 40,430 cases in males and 24,433 cases in females in 2022 [1]. Incidence is greater among males (AAR 5.7) than females (AAR 3.4), as shown in Table 2. CRC contributes to more than 4% of India’s cancer incidence and mortality. A wide disparity in regional incidence is apparent within India [2], as visualized in Fig. 1.

**Table 2:** Incidence and mortality of colorectal cancer in India, by site and sex as per GLOBOCAN 2022

		Male				Female				Total			
		Number	CR	AAR	Cum.Risk (0–74)	Number	CR	AAR	Cum.Risk (0–74)	Number	CR	AAR	Cum.Risk (0–74)
**Incidence**	Colon	21,602	3.0	3.0	0.36	12,444	1.8	1.7	0.20	34,046	2.4	2.4	0.128
	Rectum	18,828	2.6	2.6	0.31	11,989	1.8	1.7	0.19	30,817	2.2	2.2	0.25
	Colorectum	40,430	5.5	5.7	0.67	24,433	3.6	3.4	0.40	64,863	4.6	4.5	0.54
**Mortality**	Colon	13,116	1.8	1.9	0.22	7,513	1.1	1.1	0.12	20,629	1.5	1.5	0.17
	Rectum	10,843	1.5	1.5	0.18	6,895	1.0	0.96	0.11	17,738	1.3	1.2	0.14
	Colorectum	23,959	3.3	3.4	0.39	14,408	2.1	2.0	0.23	38,367	2.7	2.7	0.31

*GLOBOCAN* Global Cancer Observatory, *CR* colorectal, *AAR* age adjusted rate

The impact of urbanism on CRC incidence was marked among Indian cancer registries [2, 8]. Using data from the 12th volume of Cancer Incidence in Five Continents for 2013–17, we computed incidence rate ratios (R_a_R) between urban and rural areas of same regions. In the north and west regions, urban areas had twice the incidence of CRC than rural areas, in both sexes: Chandigarh vs. Sangrur (males:R_a_R 2.13, 95% CI 1.55–2.92; females:R_a_R 2.13, 95% CI 1.41–3.22) in Punjab and Mumbai vs. Barshi (males:R_a_R 2.18, 95% CI 1.78–2.68; females:R_a_R 2.19, 95% CI 1.75–2.74) in Maharashtra. Highest urban-rural difference was observed in north-eastern men (Kamrup urban district had almost five times greater incidence than Tripura state [R_a_R 4.71, 95% CI 3.50–6.34]) and southern women (Chennai had more than thrice the incidence of CRC than Dindigul [R_a_R 3.45, 95% CI 2.99–3.99]).

By histology, about 93% of CRCs seen at Indian hospitals were adenocarcinomas [9]. Over 45% of colon cancers and 55% of rectal cancers were diagnosed at locoregional disease stage [9].

### Age and time trends in incidence

Developed, high income regions are experiencing an apparent epidemiologic shift in CRC incidence: young/early onset’ cancer rates are escalating while overall rates are declining [10–12], especially for rectal cancer [11, 12]. This trend can be explained by screening adherence, early detection and subsequent removal of pre-cancerous lesions among people aged 55 and above [10, 11]. Early-onset CRCs are hypothesized to have a genetic and lifestyle component in their etiology [10, 13, 14]. In view of this, there have been recommendations to lower the age of screening to 45 [15]. Despite often presenting with advanced disease, they tend to have better survival [3, 16].

**Fig. 2:**
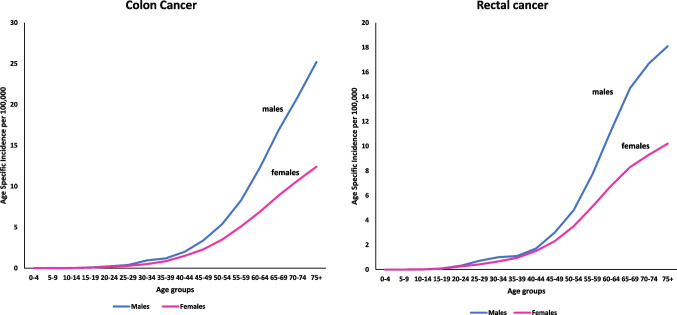
Age specific incidence curves for Colon and Rectal Cancer in India (2022); made using data from GLOBOCAN 2022: https://gco.iarc.fr/en

A general perception among Indian oncologists is that CRC presents in younger age groups in India, as compared to the west [17]. This notion is supported by data from Indian hospitals as most CRC patients visiting them are within 50-69 years [9], while a majority of CRC cases in the US and UK are of ages 65 and above [3, 18]. However, age specific incidence rates of CRC in the Indian population show no such spike among the younger ages [2], as visualized in Fig. 2. The higher number of young CRC subjects could be a reflection of the larger proportion of young population in India [17].

Currently, CRC incidence is low in India; 1 in 149 men and 1 in 250 women diagnosed in 2022 [1]. However, contrary to their developed counterparts, India and other LMICs such as Uganda and Thailand are experiencing an increasing trend of CRC [19]. Estimates from the Global Cancer Observatory suggest that CRC incidence and mortality in India shall double by 2050 [20]. This rise in incidence could be attributed to the improvement in healthcare facilities and cancer registration methodology. Changes in lifestyle and dietary practices, especially in urban regions, also contribute to this trend.

India has observed a steady rise in CRC incidence with an Annual Percentage Change (APC) of 2% to 3% for the past two decades [19]. Higher APCs were observed for colon cancer incidence in females of regions such as Chennai (5.5%), Trivandrum (10.4%), Mumbai (2.7%), Bengaluru (4%) and Pune (5.3%) [21]. In males, colon cancer incidence is increasing by 2.5%, 3.6% and 6.8% in Mumbai, Bengaluru and Dibrugarh, respectively. Rectal cancer is increasing in both sexes by 3.8% to 4.1% in Chennai, 5.9% to 7.6% in Trivandrum and 2.8% to 6.4% in Mizoram and by 4.7% in females of Kollam and 5.2% in males of Pune [21].

### Outcomes of CRC in India

Globally, India ranks fifth in CRC mortality, with 38,367 deaths in 2022 [1]. In fact, India has the second highest number of deaths due to rectal cancer, following China [1]. Mortality, like incidence, was greater for males (AAR 3.4) than females (AAR 2.0), as mentioned in Table 2. SurvCan-3 reports the five-year net survival (2008-12) for CRC in India to be 34.2% for colon cancer and 37.9% for rectal cancer [22]. Five-year relative survival of CRC in high income countries such as the US (65% for 2014–2020) and England (58.4% for 2016–2020) are higher than the survival rates of India [18, 23].

Through a comprehensive literature review, statistically significant risk factors associated with CRC have been identified, for primary prevention and risk stratification.

### Dietary factors

Considering the role of bowel mucosa in digestion, diet has been explored multiple times as an etiological factor for CRC. Consumption of red and processed meat has been heavily associated with CRC [24–28], especially in men [24, 28]. Colon cancer seemed more affected by this association than rectal cancer [26]. Associations observed were strongest in North America and weakest in Asian populations [24].

In southern India, consumption of beef quadrupled the risk of CRC (odds ratio [OR] 4.25, 95% CI 2.02–8.94) while not consuming beef showed a protective effect (OR 0.07, 95% CI 0.03- 0.19), both at high statistical significance (*p* = 0.000) [29]. Substantial increases in CRC risk were observed for consumption of eggs more than twice a week (OR 3.67 95% CI 1.23–9.35, *p* = 0.013) [30] and mutton consumption more than twice a month (OR 5.4, 95% CI 1.55–19.05, *p* = 0.008) [30].

In western India, red meat consumption was associated with more than two times elevated risk of CRC in women (OR 2.4, 95% CI 1.2–4.7) [31]. Risk increased with frequency of consumption (*p* = 0.012) [32]. Consumption of processed meat doubled the risk of CRC (OR 2.10, 95% CI 1.17–3.78, *p* = 0.013) [32].

Alternatively, choosing lean and cold meats reduced the risk of CRC in Denmark (hazard ratio [HR] 0.81, 95% CI 0.71–0.92) [33]. Consumption of fish was found to be protective against CRC [26, 29–31, 33] in general, but the dose response relationship was not significant [29]. n-3 poly unsaturated fatty acids (PUFAs) such as eicosapentaenoic acid (EPA) (RR 0.89, 95% CI 0.80–0.99) and docosahexaenoic acid (DHA) (RR 0.88, 95% CI 0.81–0.96), which are anti-inflammatory agents abundant in seafood, were associated with lower risks of CRC [34].

Consumption of fruits and vegetables is protective against CRC [29, 30, 32, 35]. The risk decreases with increasing number of servings consumed per day [25, 32, 36]. Increased consumption of solanaceous vegetables such as tomatoes (OR 0.59 95% CI 0.40–0.88), peppers (OR 0.48 95% CI 0.33–0.7) and brinjals (OR 0.42 95% CI 0.29–0.62) provided protection against CRC probably due to possible anti-cancer agents such as pectin, anthocyanins, glycosidic alkaloids and carotenoids [36]. In an Indian study, cabbage consumption reduced the risk of developing CRC by half (OR 0.5, 95% CI 0.3–0.8) [31]. Daily consumption of fruits and vegetables reduced CRC risk by about 90% in another Indian study [32]. This could be attributed to dietary fibre which is a well explored protective agent against CRC [36]. Contrarily, increased consumption of starchy vegetables such as potato (OR 1.76; 95% CI: 1.26–2.47) [36] and tapioca (OR 2.70, 95% CI 1.32, 3.31, *p* = 0.000) [29], has been associated with an elevated risk of developing CRC.

Thirteen studies on nuts consumption found it to be protective against CRC, especially in Asia (RR 0.44, 95% CI 0.29–0.68) [37]. Twenty-nine studies concluded that CRC risk and legume consumption are inversely associated (RR 0.90, 95% CI 0.83–0.98) [37]. Increment in servings of nuts and legumes brought about a risk reduction of 21% to 33%. Consumption of whole grains is also protective against CRC [27].

Meta-analysis of multiple cohort studies revealed that higher intake of dairy products was protective against CRC with a significant dose response relationship [38]. It also reduced CRC mortality [38]. The association was significant for milk [38] and for fermented dairy products, including cheese and yoghurt [39] and stronger in Europe [38, 39].

The protective effect of vitamins has been demonstrated to cause 12% to 25% reduction against CRC in multiple meta-analyses [36, 40–43]. Risk reduction was stronger in women and further reduced with increasing dosage [41–43]. Multivitamin supplements (RR 0.92, 95% CI 0.87–0.97) were also protective against CRC [42].

Among minerals, calcium is a well-established protective agent against CRC [36], that may be involved in the protective effect extended by dairy products. About 16% of disability-adjusted life-years (DALYs) caused by CRC in South Asia have been attributed to diets low in calcium [4]. Calcium supplements significantly decreased CRC risk (RR 0.86; 95% CI 0.79,0.95) [42].

A pro-inflammatory diet which is calorie dense but deficient in dietary fibre caused a 40% increase in risk of CRC (RR 1.40, 95% CI 1.26–1.55, *p* < 0.001) [44], especially in men. Processed diet pattern (including confectioneries and fast food) in Malaysia increased the risk of CRC by over three times (OR 3.45, 95% CI 1.25–9.52, *p* = 0.017) [45]. Daily and incremental intake of sugary beverages is positively associated with CRC incidence and mortality, especially among the physically inactive and obese [46]. Intake of **fried **food more than twice a month doubled the risk of CRC in a south Indian case-control study (OR 2.03, 95% CI 0.95–4.43, *p* = 0.06) [30].

Having a plant based diet was protective against CRC in the US, especially if ultra-processed elements such as refined sugars or flour were avoided [47]. Similarly, the micronutrient-rich Mediterranean diet showed a significant reduction in CRC risk in Italy. However, no single dietary component was attributed individually for the risk reduction [48]. This emphasises the importance of a well-balanced diet to bring about risk reduction. Adherence to dietary guidelines that promote such well-balanced and healthy diets has been observed to reduce CRC risk by half [33].

### Behavioral factors

**Physical activity** is a protective factor supported by multiple cohort studies [5, 25, 35]. A meta-analysis of 18 studies found a 38% lower risk of CRC among physically active subjects (RR 0.63, 95% CI 0.47–0.84) [49]. Risk was lower among participants with low BMI and no family history of CRC.

Strong evidence from multiple studies supports that regular and greater intake of alcohol is associated with elevated risk of CRC [5, 27, 32, 50], with stronger association in men [50]. Longer duration in years and higher intake further increased risk in men [50].

Smoking was positively associated with CRC [5, 25, 50]. In Indian case-control studies, ORs for tobacco smoking were 2.77 in the west and 8.79 in the south [29, 32]. The risk increased significantly with amount and duration of cigarettes smoked [25, 32, 50]. Even passive smoking had an evident association with CRC (RR 1.14; 95% CI 1.05–1.24) [51], especially in males.

### Genetic factors

An analysis of 19 studies found a 14% increase in risk with every 10-cm increase in height (HR 1.14, 95% CI 1.11–1.17, *p* < 0.001), with greater risk for colon cancer [52]. Postulated attributions for this association are increased body organ size due to greater stature and related hormonal and genetic factors affecting carcinogenesis [52].

Family history of CRC was significantly associated with an increased risk of CRC [5, 13, 14, 25], especially early onset CRC [13]. This risk increased further with genetic closeness of the relative such that having first degree relatives with CRC doubled the risk [13]. Even the presence of colorectal polyps or adenomas in first degree relatives was found to increase CRC risk by about 40% (OR 1.40, 95% CI 1.35–1.45) [14]. A 16 fold increase in risk for early onset CRC was observed among those having two or more first degree relatives with both polyps and CRC (OR 16.57, 95% CI 4.81–57.13) [14]. Thus, the hereditary component in early onset CRC is significantly stronger.

A meta-analysis of 14 studies found the presence of BRCA1 mutation to be a risk factor for CRC (OR 1.58, 95% CI 1.23–1.98, *p* < 0.001) [53].

### Colorectal cancer as a second primary

**History of cancers** such as testicular or prostate increased the risk of developing CRC by 44% in UK (HR 1.44, 95% CI 1.01–2.08, *p* = 0.05) [5]. Previous history of cervical cancer (HR 1.20, 95% CI 1.03–1.40, *p* < 0.05), endometrial cancer (HR 2.26, 95% CI 1.77–2.90, *p* < 0.001) or ovarian cancer (HR 2.09, 95% CI 1.59–2.76, *p* < 0.001) was positively associated with CRC [54].

Reproductive cancers share risk factors such as obesity and lifestyle factors with CRC. Besides that, radiation exposure during cancer treatment could also be responsible for new incidence. Endometrial cancer patients who received radiotherapy, especially external beam radiotherapy, had higher risk of CRC [54], significant for both colon (*p* < 0.001) and rectum (*p* = 0.023) [55]. The cumulative incidence for colon cancer at 30 years was 2.5% for patients receiving no radiation and 3.7% for patients receiving any radiation (*p* < 0.001) [55]. Subdiaphragmatic radiotherapy caused an increase of CRC incidence in survivors of Hodgkin lymphoma (R_a_R 3.5, 95% CI 1.0–11.5) [56]. Incremental doses of radiation on large bowel increased the risk (*p* < 0.001) [56]. There was no such effect observed for supra-diaphragmatic radiation. This indicates a need for protective measures to minimize the effect of radiation on healthy tissue and further research in the area.

Ovarian cancer patients who underwent surgery had greater risk of developing CRC than those left untreated (HR 3.79, 95% CI 1.11–12.9, *p* < 0.05) [54]. A part of this hazard could be attributed to the possibility of incidental diagnosis during surgery. Ovarian and endometrial cancer patients who received chemotherapy had higher risk of developing CRC [54]. The hazards of developing CRC were greater for young gynecological cancer patients, particularly during the first three years of follow-up [54].

### Illnesses and infections

Features of obesity such as increase in body mass index (BMI) and waist circumference have been positively associated with CRC [25, 32], especially in males (RR 1.29 per 8 kg/m^2^ increase, 95% CI 1.26–1.34) [25]. Childhood obesity, that remained unresolved in adulthood, further increased the risk of developing CRC, especially of colon cancer [57]. A Canadian study attributed the increasing incidence of young onset CRC to increasing patterns of obesity [10].

Diabetes mellitus was significantly associated with CRC in multiple populations [5, 32, 35, 58], especially in females, with higher risk for rectal cancer than colon cancer [58]. Long-term diabetics have increased risk for both incidence and mortality [5]. Glaucoma patients also had higher CRC risk (HR 1.36, 95% CI 1.06–1.74, *p* = 0.02) [5]. History of heart failure doubled the risk of CRC in UK (HR 1.96, 1.13–3.40, *p* = 0.02) [5]. Having metabolic syndrome, characterized by abnormalities in waist circumference, BMI, hypertension, hyperlipidemia and blood sugar, doubled the risk of CRC in females (HR 2.23, 95% CI 1.32–3.77, *p* = 0.003) [59]. The role of sugar and lipid metabolism in CRC development is thus apparent.

Patients of hemorrhoids experienced greater incidence of CRC in a Taiwanese cohort, especially young patients below 50 years (HR 3.53 95% CI 2.15–5.79), specifically rectal cancer [60]. Irritable bowel syndrome (IBS) (OR 2.8, CI 2.305–3.294) was associated with higher CRC risk, particularly within the first year of diagnosis [61]. The association is likely due to similarity of symptoms with CRC.

A meta-analysis verified that having inflammatory bowel disease (IBD) (RR 2.93 95% CI 1.79–4.81) tripled the risk of developing CRC [25]. The prevalence of CRC among ulcerative colitis patients in an Indian cohort was 1.9% [6]. The cumulative risk of developing CRC was 1.5% in the first, 7.2% in the second and 23.6% in the third decade of follow-up among such patients [6]. Changes in the gut microbiome cause inflammation that gives rise to conditions such as IBD and colitis and thus, enables carcinogenesis [62].

***Helicobacter pylori*** is a known risk factor for gastric cancer; however, it was correlated with CRC (OR 1.58, 95% CI 1.05–2.37) [63]. This was in a meta-analysis of Chinese studies, which therefore, has limited generalizability. Other bacterial infections have also found significance in CRC etiology research, albeit in smaller sample size studies. Certain studies are recommended for Indian population.

HPV prevalence in CRC patients from China was found to be 0.45 [64]. With an OR of 10.78 (95% CI 4.22–27.53), a meta-analysis found HPV infection significantly associated with CRC. Odds were greater for rectal cancer (OR 8.41, 95% CI 5.5–12.86) than colon (OR 4.28, 95% CI 2.64–6.93) [64]. HPV-16 was more prevalent than HPV-18 in these patients.

The prevalence of human cytomegalovirus (HCMV) (also known as human herpes virus-5) was 27.5% among CRC patients, ranging from 3.6% in the US to 42% in Asia [65]. Having HCMV infection caused a sixfold elevated risk of CRC (OR 6.59 95% CI 4.48–9.69) [65]. Developed regions had 74% lesser odds of developing CRC due to HCMV than developing nations (OR 0.26, 95% CI 0.16–0.41) [65].

CRC patients were more likely to be positive for hepatitis B virus (HBV) than controls (OR 1.27, 95% CI 1.20–1.33) [66]. Risks were similar for men and women but higher for colon cancer. Among HBV positive individuals, younger patients (OR 1.63, 95% CI 1.48–1.79) had a significantly higher risk than older patients (OR 1.24, 95% CI 1.13–1.37) (*p* < 0.001) [66].

Lynch syndrome or hereditary non-polyposis colorectal cancer syndrome is a genetic condition which predisposes colorectal carcinogenesis. It is the most common hereditary condition in early-onset CRC [67]. Estimated prevalence of CRC in individuals with Lynch syndrome is 2.2%, ranging from 0.4% to 21.2%, using 51 studies from 18 countries. Studies that excluded patients above 75 years had double the prevalence (4.4%) [67]. Other hereditary conditions that have a huge lifetime risk of developing CRC include familial adenomatous polyposis, MUTYH-associated polyposis and juvenile polyposis syndrome [7]. Hereditary CRC syndromes had a prevalence of 1.9% among CRC cases seen at a leading Indian tertiary cancer care centre [17].

### Pharmaceutical factors

Metformin use was found to be protective against CRC (OR 0.75, 95% CI 0.62–0.91, *p* = 0.004) [35] but without a dose response relationship. No other antidiabetic drug was associated with CRC [35].

Compared to non-users of non-steroidal anti-inflammatory drugs (NSAIDs), daily use of aspirin was found to decrease CRC risk by 30% to 40% in case-control studies [35]. However, this association was not significant in cohort studies [25].

Any antibiotic use was associated with an increased risk of CRC (for early onset CRC: OR 1.49, 95% CI 1.07–2.07, *p* = 0.018, for older patients: OR 1.09, 95% CI 1.01–1.18, *p* = 0.029) [68] but no dose response relationship was observed.

### Environmental and occupational exposures

Largely, industrial professions including manufacturing professions were significantly associated with a higher risk of CRC (RR 1.12, 95% CI 1.03–1.23) [69]. Particularly those involved in manufacture of leather products (RR 1.7, 95% CI 1.24–2.34), basic metals (RR 1.32, 95% CI 1.07–1.65), electronic and optical products (RR 2.14, 95% CI 1.04–4.50), machinery and equipment (RR 2.2 95% CI 1.03–4.72) had higher risks of developing CRC [69]. Asbestos exposure is attributed for higher risk of CRC among shipyard workers (RR 1.40, 95% CI 1.07–1.84) in Italy [69].

Psychological stress was positively associated with rectal cancer, men had about two times and women had two to three times increased risk at moderate to high levels of perceived stress [70]. Night-shift workers had a 32% higher risk of CRC as per a meta-analysis of six studies from western countries (OR 1.63, 95% CI 1.32–2.01) [71], perhaps due to the long-term disruption of the circadian rhythm.

A Korean cohort study found positive associations between serum concentration of organochlorine pesticides and CRC, especially in higher concentrations of trans-nonachlor (HR 4.86, 95% CI 1.95–12.16) and p,p-Dichlorodiphenyldichloroethane (HR 7.43, 95% CI 2.42–22.84) and polychlorinated biphenyls [72]. Insecticides such as Aldicarb and Fonofos were significantly associated with colon cancer, while Carbaryl, Chloropyrifos and Toxophene augmented the risk of rectal cancer [72, 73]. 

**Fig. 3:**
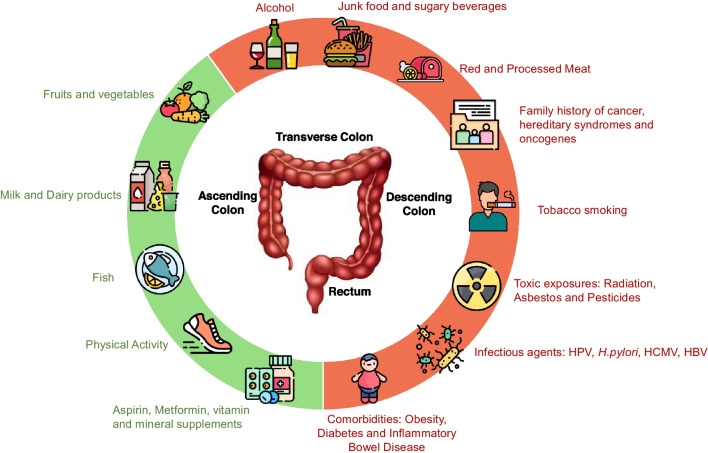
Risk factors of colorectal cancer identified in this review; Open access icons used in this figure were downloaded from https://www.freepik.com

Risk factors of CRC have been summarized as Fig. 3.

### Overview of studies

Colorectal cancer rates are increasing annually in India, posing a precarious public health challenge in the coming years. Poor access to healthcare and high risk factor prevalence, combined with rapid globalization, stimulate this cancer burden. This review was aimed at addressing this emergent issue by providing recent and relevant statistics.

This review describes results from more than 50 studies worldwide, which have been bifurcated by study methodology and sample size in Table 3. Currently, evidence from India is restricted to case-control and cross-sectional studies, most of which are small sample hospital-based studies. Results of studies based on small sample size have limitations; they are often subject to bias and need careful consideration. A population as vast and diverse as India needs more research, given the complicated and multifactorial etiology of CRC. For example, southern and north-eastern registries in India report high incidence of CRC [2], with a significantly increasing trend of incidence [21], possibly linked to the higher prevalence of meat consumption in these regions [74]. Interestingly, these states also report higher consumption of fish [74], which is evidently protective against CRC in case-control studies. Meanwhile, low incidence of CRC in India has often been credited to the higher prevalence of vegetarianism. Cohort studies are warranted to understand the role of complex dietary patterns practiced in India in the development of CRC.

**Table 3:** Modifiable and Non-Modifiable risk factors identified through literature review

Risk factors	Association	Case–control studies		Cohort studies		Meta-analyses	
		200 cases or less	More than 200 cases	Less than 500 cases	More than 500 cases	20 studies or less	More than 20 studies
*Modifiable risk factors:*							
1. **Diet**							
• Red and processed meat	Positive	[29, 30, 32]	[31]		[26, 28]		[24, 25, 27]
• Fish and fish products	Inverse	[29, 30]	[31]		[26]		
• Milk and dairy products	Inverse					[39]	[38]
• Fruits and vegetables (dietary fibre and antioxidants)	Inverse	[29, 30, 32]	[35, 36]	[48]	[47]		[25]
• Starchy vegetables (potatoes, tapioca)	Positive	[29]	[36]				
• Unhealthy food (processed and fried food, sugary beverages)	Positive	[29, 30, 45]			[46]	[44]	
• Whole grains, nuts and legumes	Inverse						[27, 37]
• Vitamins and minerals (through diet/supplement)	Inverse		[36]			[40]	[41–43]
2. **Behavioral and Lifestyle factors**							
• Tobacco/smoking	Positive	[29, 32]		[50]	[5]	[25, 51]	
• Alcohol	Positive			[50]	[5]		[27]
• Physical activity	Inverse		[35]		[5]	[49]	[25]
3. **Pharmaceutical factors**							
• Metformin	Inverse		[35]				
• Aspirin	Inverse		[35]				[25]
• Antibiotics	Positive		[68]				
4. **Environmental and Occupational factors**							
• Asbestos	Positive						[69]
• Manufacturing professions	Positive						
• Stress	Positive				[70]		
• Night Shift	Positive						
• Pesticides	Positive	[72]		[73]			
*Non-Modifiable risk factors:*							
5. **Disorders**							
• Obesity	Positive	[32]				[57]	[25]
• Diabetes	Positive	[32]	[35]	[58]	[5]		
• Metabolic syndrome	Positive			[59]			
• Inflammatory bowel disease	Positive			[6]			[25]
• Irritable bowel syndrome	Positive					[61]	
• Hemorrhoids	Positive			[60]			
6. **Infections**							
• Bacterial: *H. pylori*	Positive					[63]	
• Viral: HPV, HCMV, HBV	Positive		[66]			[64, 65]	
7. **Familial, Genetic and Hereditary factors**							
• Family history of colorectal cancer/adenoma	Positive		[14]		[5, 13]		[25]
• Personal history of cancer (especially of reproductive organs) • Treatment of cancer (radiation, chemotherapy, surgery)	Positive	[56]		[55]	[5, 54]		
• Hereditary colorectal cancer syndromes such as Lynch syndrome and familial adenomatous polyposis	Positive	[17]					[67]
• Height	Positive						[52]
• BRCA mutation	Positive					[53]	

*HPV* human papilloma virus, *HCMV* human cytomegalovirus, *HBV* hepatitis B virus, *BRCA* breast cancer gene

Early-onset CRC is gaining prevalence in the developed world. In India, changing dietary patterns with rapid globalization pose a threat to our young demography. While young-onset CRC is privy to genetic and obesity related factors, older onset CRC is affected by social deprivation and underprivileged settings. Despite having markedly lower incidence rates than urban populations, rural and socio-economically underprivileged groups experience poor disease prognosis. Rurality and social privilege are important factors affecting survival, besides treatment completion, disease stage and patient age [16].

Nearly three-quarters of global CRC burden stems from modifiable risk factors [4]. The International Agency for Research on Cancer (IARC) has classified alcohol, processed meat and tobacco smoking within Group I “Carcinogenic to humans” for CRC [75]. Red meat, night shift work, firefighting as an occupation and Asbestos are considered as Group 2A “Probable Carcinogens” for CRC with limited evidence in humans [75]. X and gamma radiation is mentioned within group I for colon cancer but group 2A for rectal cancer. Evidence for other factors is currently limited and need more conclusive studies.

### Recommendations

To enable the primary prevention of CRC, education regarding risk factors of CRC to general physicians and health workers combined with health education of general population to raise risk factor awareness is essential. Promoting consumption of balanced diets with high fibre intake, reducing red meat consumption, maintenance of healthy body weight and curbing tobacco and alcohol use can potentially reduce CRC incidence in addition to other health benefits.

Screening for CRC is widely practiced in many western countries. The tests used include fecal tests or direct visualization depending on the patient’s risk stratification, availability of resources and preferences [15, 76, 77]. Stool-based tests such as guaiac fecal occult blood tests (gFOBTs) (sensitivity: 50% to 75%, specificity: 96% to 99%) and fecal immunochemical tests (FITs) (sensitivity: 74% to 81%, specificity: 95%) are non-invasive and low-cost with high specificity [77]. Direct visualization tests such as colonoscopy (sensitivity: 95%, specificity: 86% to 89%) and sigmoidoscopy (sensitivity: 95%, specificity: 87%), on the other hand, are resource intensive, need trained personnel but have better sensitivity and proven efficacy in reducing CRC mortality in multiple cohorts and trials [77]. The United States Preventive Services Task Force has recommended several screening options including screening by fecal tests (FITs/FOBTs) annually, sigmoidoscopy every five years or colonoscopy at 10 years interval for adults starting at age 50 years and continuing until age 75 years [15].

To conclude, colorectal cancer rates in India are on the rise, particularly in men and in urban and older populations. Red meat and ultra-processed food consumption, alcohol abuse and tobacco smoking are significant yet controllable risk factors of CRC. Other risk factors include personal and family history of cancer/adenomas, co-morbidities such as diabetes and IBD, pesticide exposure and previous cancer treatment. Protective factors include a well-balanced diet high in fibre, rich in dairy products and fish, adequate intake of calcium and vitamins and drugs including metformin and aspirin.

Considering the Indian situation, where incidence rates are not high and there are limited resources in the healthcare system, population-based mass screening for CRC may not be cost-effective and is currently not recommended. Yet, persons at-risk i.e. those with personal or family history of IBD, adenomas, CRC and hereditary conditions such as Lynch syndrome, must be offered opportunistic high-risk screening [5].

## Data Availability

The data and articles accessed and reviewed in this paper is available on public domain either on open access or by subscription.

## Declarations

### Disclaimer

The authors are solely responsible for the data and the content of the paper. In no way, the Honorary Editor-in-Chief, Editorial Board Members, the Indian Society of Gastroenterology, printer/publishers, or are responsible for the results/findings and content of this article.
